# Supplementary material for: Altered Lipid Moieties and Carbonyls in a Wistar Rat Dietary Model of Subclinical Fatty Liver: Potential Sex-Specific Biomarkers of Early Fatty Liver Disease?
Source: Antioxidants (Basel). 2023 Sep 28;12(10):1808. doi: 10.3390/antiox12101808 (PMC10604774; doi:10.3390/antiox12101808)
Supplement: Supplementary file 1 [file antioxidants-12-01808-s001.zip › Supplementary Materials_Martin-Grau.M_220823.pdf]

Supplementary Materials

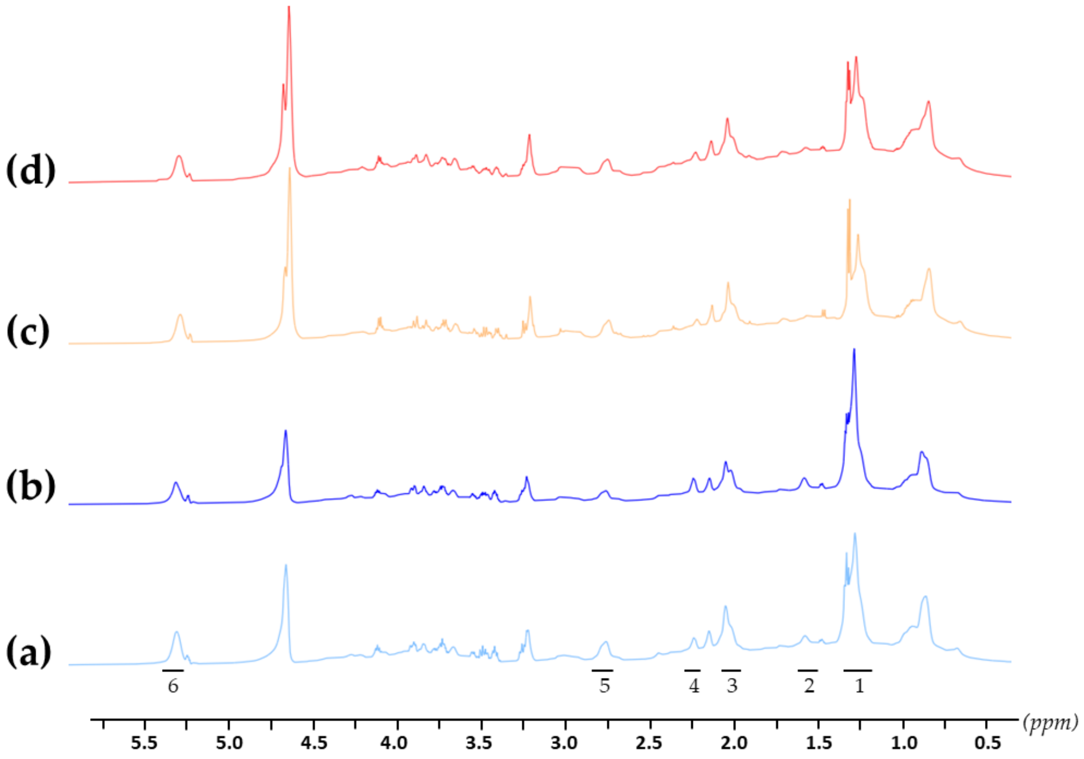

Figure S1. Average serum spectra by ^{1}H-NMR in Wistar rats. Each spectrum represents the mean spectrum of each experimental group being (**a**), CTL males; (**b**), HFD males; (**c**), CTL Females; (**d**), HFD Females. Lipid regions are represented from 1 to 6. 1, Saturated fatty acids (SFA); 2, Long Chain Carbonyl groups (lcCO); 3, Long chain Unsaturated fatty acids (lcUFA); 4, Total Carbonyl groups (tCO); 5, Polyunsaturated fatty acids (PUFA); 6, total UFAs (tUFA).

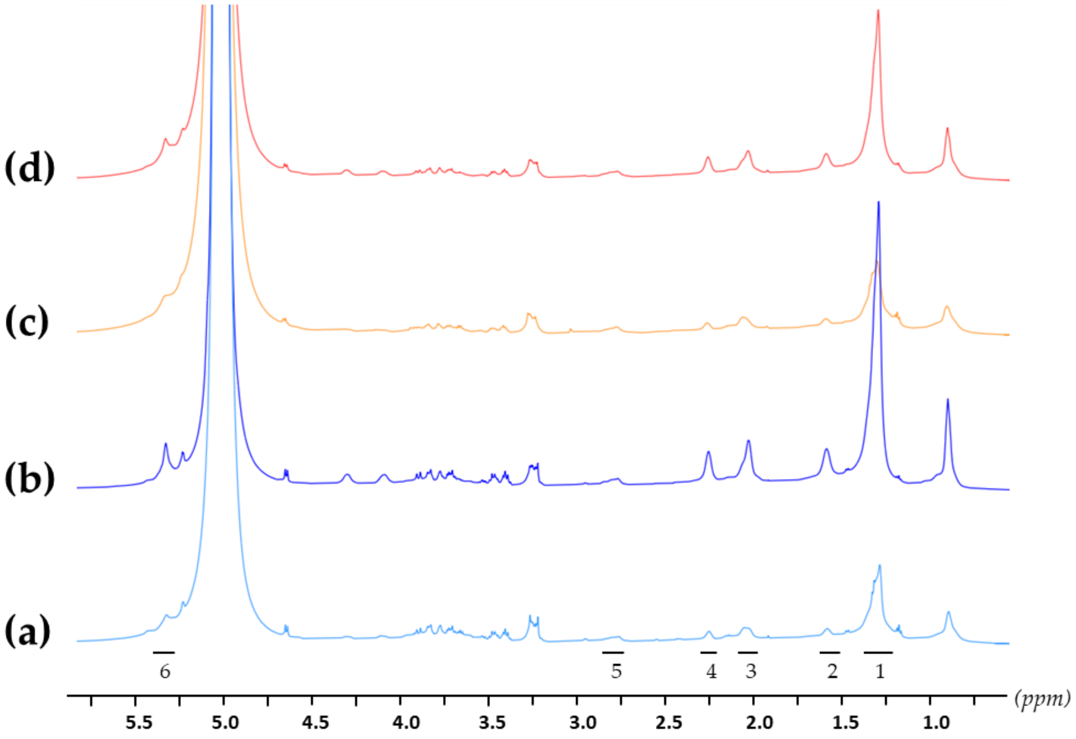

Figure S2. Average liver spectra by HR-MAS ^{1}H-NMR in Wistar rats. Each spectrum represents the mean spectrum of each experimental group being (**a**), CTL males; (**b**), HFD males; (**c**), CTL Females; (**d**), HFD Females. Lipid regions are represented from 1 to 6. 1, Saturated fatty acids (SFA); 2, Long Chain Carbonyl groups (lcCO); 3, Long chain Unsaturated fatty acids (lcUFA); 4, Total Carbonyl groups (tCO); 5, Polyunsaturated fatty acids (PUFA); 6, total UFAs (tUFA).

Table S1. Lipid regions values in liver and serum at end point by metabolomics in Wistar rats.

**Parameter**		**Males**		**Females**	
		**CTL** (*n*=8)	**HFD** (*n*=8)	**CTL** (*n*=8)	**HFD** (*n*=7)
Liver	1	26,17 ± 1,17	34,11 ± 1,42***	23,74 ± 1,73†	30,50 ± 2,14***††
	2	10,59 ± 0,83	12,43 ± 0,84*	9,80 ± 0,84	11,02 ± 0,96*
	3	6,27 ± 0,38	8,18 ± 0,39***	5,56 ± 0,37††	6,92 ± 0,39***†††
	4	6,34 ± 0,91	8,43 ± 0,69**	5,41 ± 1,01	7,01 ± 1,06*†
	5	8,24 ± 1,42	6,30 ± 0,42**	7,04 ± 0,91	6,67 ± 0,60
	6	24,72 ± 4,40	14,36 ± 2,57***	32,78 ± 4,85††	22,53 ± 4,03***††
Serum	1	30,85 ± 1,64	35,91 ± 2,24**	27,67 ± 2,83	28,39 ± 2,90†††
	2	10,78 ± 0,36	10,87 ± 0,29	11,57 ± 1,00	12,15 ± 1,03
	3	7,40 ± 0,38	7,43 ± 0,30	8,05 ± 0,28††	8,00 ± 0,29†
	4	6,47 ± 0,39	6,90 ± 0,18	6,20 ± 0,39	6,75 ± 0,39*
	5	8,40 ± 0,38	6,45 ± 0,60***	8,33 ± 0,41	8,03 ± 0,50†††
	6	9,04 ± 0,67	7,23 ± 0,82***	7,09 ± 0,78†††	6,24 ± 0,66

Data are expressed as the mean values ± standard deviation of the different experimental groups (CTL males *n*=8; HFD males *n*=8; CTL females *n*=8, HFD females *n*=7). Significant differences calculated by ANOVA and post hoc test and non-parametric Kruskal-Wallis. *p_value<0,05; **p_value<0,01; ***p_value<0,001 between CTL and HFD groups; †p_value<0,05; ††p_value<0,01; †††p_value<0,001 between CTL males and CTL females or HFD males and HFD females. Abbreviations: 1, Saturated fatty acids (SFA); 2, Long Chain Carbonyl groups (lcCO); 3, Long chain Unsaturated fatty acids (lcUFA); 4, Total Carbonyl groups (tCO); 5, Polyunsaturated fatty acids (PUFA); 6, total UFAs (tUFA).

Table S2. Human cohort composition and comparison of metabolic characteristics (profile and components) in the entire cohort (Total), and in subsets of women and men patients.

**Variables**	**All** (*n* = 264)	**Males** (*n* = 72)	**Females** (*n* = 192)
Age (years)	54,5 ± 14,4	52,4 ± 13,4	56,4 ± 15,6
BMI (kg m−2)	48,7 ± 6,5	48,3 ± 7,6	47,6 ± 6,8
HOMA-IR	3,3 ± 2,7	3,6 ± 2,8	3,0 ± 2,3
s-Glucose (mg/dL)	102 ± 35	102 ± 34	100 ± 37
s-LDL Cholesterol (mg/dL)	120 ± 35	121 ± 32	125 ± 39
s-HDL Cholesterol (mg/dL)	44 ± 14	36 ± 10	42 ± 15
s-Triglycerides (mg/dL)	147 ± 64	153 ± 62	139 ± 60
ALT (U/L)	23 ± 10	27 ± 10	19 ± 7
TBIL (mg/dL)	0,72 ± 0,34	0,78 ± 0,34	0,67 ± 0,34

Abbreviations: ALT, alanine aminotransferase; BMI, body mass index; HDL, high-density lipoprotein; LDL, low-density lipoprotein; TBIL, total bilirubin.
