# Supplementary figures and images for: Altered Lipid Moieties and Carbonyls in a Wistar Rat Dietary Model of Subclinical Fatty Liver: Potential Sex-Specific Biomarkers of Early Fatty Liver Disease?
Source: Antioxidants (Basel). 2023 Sep 28;12(10):1808. doi: 10.3390/antiox12101808 (PMC10604774; doi:10.3390/antiox12101808)

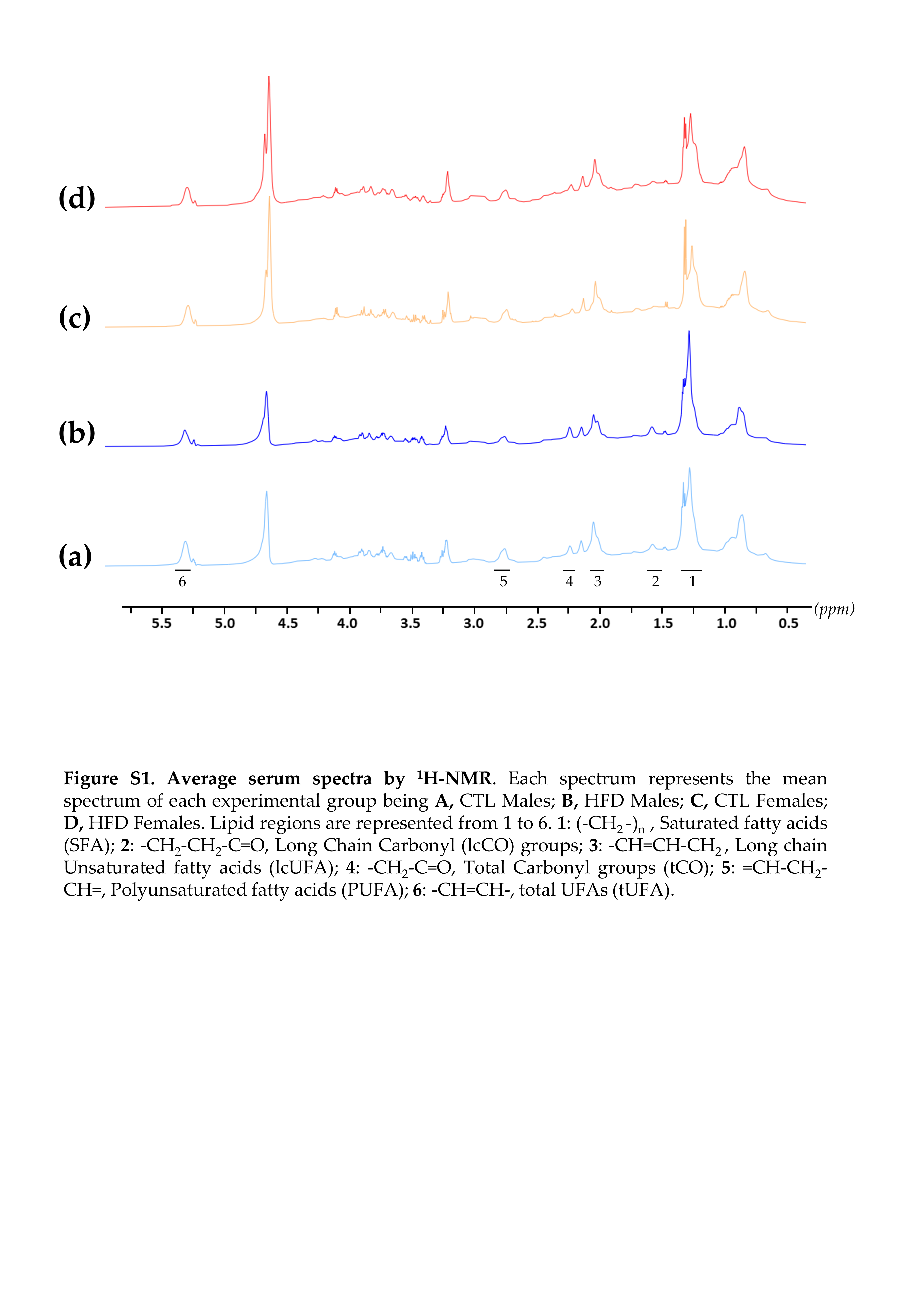

Supplement: Supplementary file 1 [file antioxidants-12-01808-s001.zip › Diapositiva6.TIF]

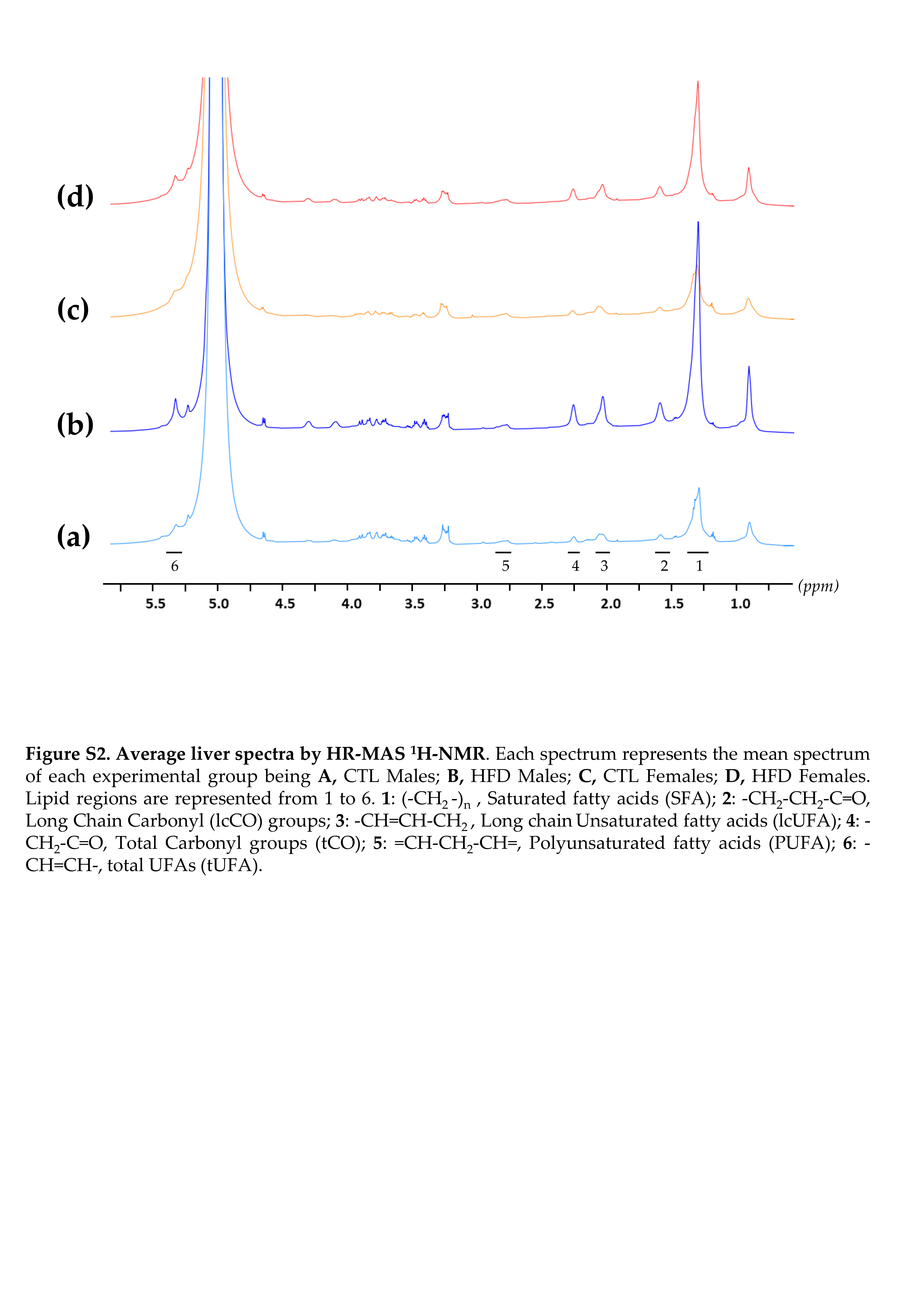

Supplement: Supplementary file 1 [file antioxidants-12-01808-s001.zip › Diapositiva7.TIF]
